# Determinants of Meal-Induced Changes in Circulating FFA Epoxides, Diols, and Diol-to-Epoxide Ratios as Indices of Soluble Epoxide Hydrolase Activity

**DOI:** 10.3390/ijms242417351

**Published:** 2023-12-11

**Authors:** Young Taek Oh, Jun Yang, Darko Stefanovski, Bruce Hammock, Jang H. Youn

**Affiliations:** 1 Department of Physiology and Neuroscience, University of Southern California Keck School of Medicine, Los Angeles, CA 90089, USA; youngoh@usc.edu; 2 Department of Entomology and Nematology, Comprehensive Cancer Center UCDMC, University of California, Davis, CA 95616, USA; junyang@ucdavis.edu (J.Y.); bdhammock@ucdavis.edu (B.H.); 3 Department of Biostatistics, University of Pennsylvania School of Veterinary Medicine, Philadelphia, PA 19146, USA; sdarko@vet.upenn.edu

**Keywords:** liver, heart, renal excretion, dietary oxylipins, urine

## Abstract

Soluble epoxide hydrolase (sEH) is an important enzyme for metabolic and cardiovascular health. sEH converts FFA epoxides (EpFAs), many of which are regulators of various cellular processes, to biologically less active diols. In human studies, diol (sEH product) to EpFA (sEH substrate) ratios in plasma or serum have been used as indices of sEH activity. We previously showed these ratios profoundly decreased in rats during acute feeding, possibly reflecting decreases in tissue sEH activities. The present study was designed to test which tissue(s) these measurements in the blood represent and if factors other than sEH activity, such as renal excretion or dietary intake of EpFAs and diols, significantly alter plasma EpFAs, diols, and/or their ratios. The results show that postprandial changes in EpFAs and diols and their ratios in plasma were very similar to those observed in the liver but not in other tissues, suggesting that the liver is largely responsible for these changes in plasma levels. EpFAs and diols were excreted into the urine, but their levels were not significantly altered by feeding, suggesting that renal excretion of EpFAs and diols may not play a major role in postprandial changes in circulating EpFAs, diols, or their ratios. Diet intake had significant impacts on circulating EpFA and diol levels but not on diol-to-EpFA (D-to-E) ratios, suggesting that these ratios, reflecting sEH activities, may not be significantly affected by the availability of sEH substrates (i.e., EpFAs). In conclusion, changes in FFA D-to-E ratios in plasma may reflect those in the liver, which may in turn represent sEH activities in the liver, and they may not be significantly affected by renal excretion or the dietary intake of EpFAs and diols.

## 1. Introduction

Oxylipins, oxygenated lipids abundant in aerobic organisms, are involved in various cellular signaling pathways [1,2,3]. Oxylipins are derived from unsaturated free fatty acids (FFAs) by three enzyme families: cyclooxygenases (COX), lipoxygenases (LOX), and cytochromes P450 (CYP). Among these oxylipins, FFA epoxides (EpFAs), produced by multiple CYPs, bring about largely beneficial effects on metabolic and cardiovascular health [4,5,6]. EpFAs are metabolized to diols by epoxide hydrolases, mainly, but not exclusively, by the soluble epoxide hydrolase (sEH). The inhibition of sEH increases EpFAs, enhancing the beneficial effects associated with EpFAs [4,5,6,7]. The significant association of sEH polymorphism with various metabolic or cardiovascular diseases [8,9,10,11,12] suggests an important role for sEH biology in human diseases. sEH inhibition is proposed as an effective treatment option for metabolic and cardiovascular diseases [1,7]. Recent studies also showed the potential of sEH inhibition in the treatment of lung, kidney, and neural diseases [13,14]. 

**Figure 1:**
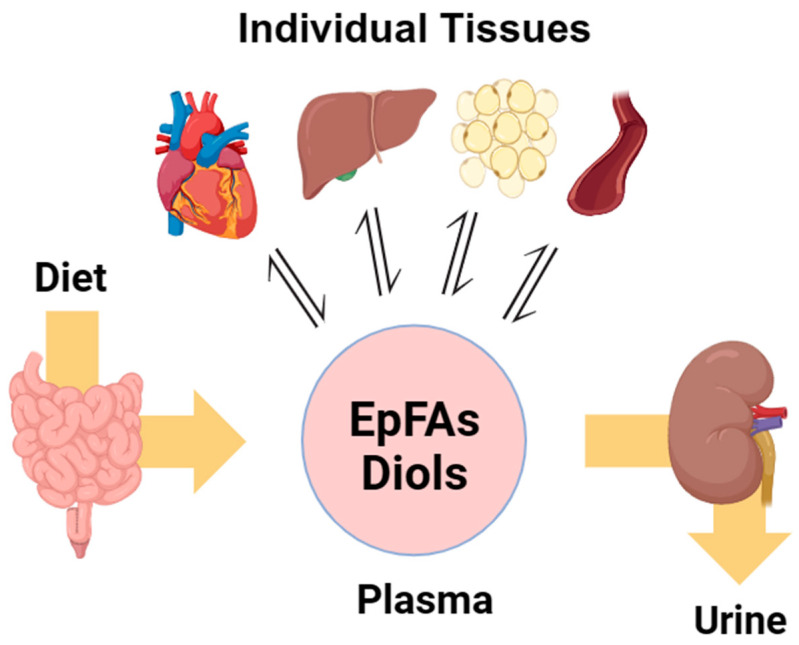
A schematic diagram illustrating the concept that diets, individual tissues, and renal excretion can influence plasma EpFA and diol levels. Figure was created with BioRender.com.

Despite its importance in human physiology and health, sEH is difficult to study in humans because of limited access to the individual tissues where sEH is expressed. Therefore, in human studies, the ratios of diol (sEH product) to EpFA (sEH substrate) in plasma or serum have been popularly used as indices of sEH activity [12,13,14]. However, it is unknown which tissues these measurements in the blood represent; enzymes involved in oxylipin metabolism exist in individual tissues or cells but not in blood plasma. Plasma oxylipin levels may be largely determined by exchanges of oxylipins between plasma and individual tissues (Figure 1), which may have quite different oxylipin profiles, depending on FFA availability and enzyme activities for oxylipin metabolism in these tissues. Plasma oxylipins or diol-to-EpFA (D-to-E) ratios may represent an average of those in all individual tissues. Alternatively, they may represent those in specific tissues that predominantly affect plasma-oxylipin levels. The direct release and reuptake of EpFAs and possibly diols into membrane phospholipids or lipoproteins also warrants study. It is also important to test if factors other than sEH activity alter plasma EpFA and diol levels and their ratios [4,11]. For example, EpFAs and diols and their conjugates are excreted to the urine, and changes in urinary EpFA and diol excretions may alter circulating EpFAs, diols, and D-to-E ratios in the blood [15]. Furthermore, dietary oxylipins may alter circulating EpFAs, diols, and D-to-E ratios [16]. These critical issues have not been directly addressed, despite the increasing use of D-to-E ratios as the indices of sEH activity in human studies [17,18,19,20].

Our previous study [21] showed that plasma D-to-E ratios profoundly decreased after a meal, possibly reflecting decreases in tissue sEH activities. One goal of the present study was to examine whether these postprandial decreases in D-to-E ratios are similarly observed in all or specific tissues; we determined changes after acute feeding in plasma and individual-tissue oxylipins to address the issue of which tissue(s) is responsible for changes in plasma EpFAs, diols, and D-to-E ratios. Another goal of the present study was to evaluate the possible role of the kidneys in altering plasma EpFAs, diols, and D-to-E ratios; we tested if renal excretions of EpFAs or diols are altered after acute feeding to alter plasma D-to-E ratios independent of changes in tissue sEH activity. Finally, we studied the effects of grain-based natural vs. casein-based purified diets to evaluate the impact of dietary oxylipins on circulating EpFAs, diols, and D-to-E ratios, with the expectation that grain-based diets contain more oxylipins than casein-based purified diets. 

## 2. Results

### 2.1. Effects of Acute Feeding on EpFAs, Diols, and D-to-E Ratios in Plasma and Individual Tissues

**Figure 2:**
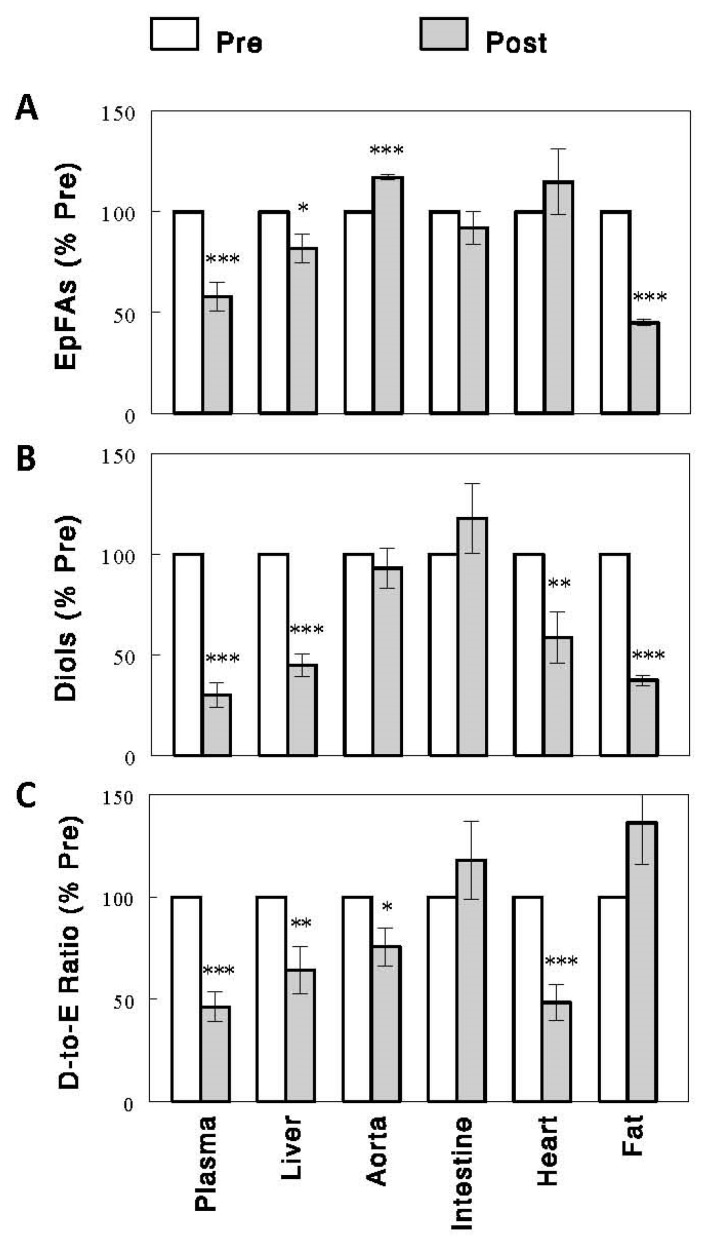
EpFAs (**A**), diols (**B**), and D-to-E ratios (**C**) in plasma and individual tissues before (preprandial state, Pre) and after (postprandial state, Post) on acute, 2.5 h feeding in rats. Data are means ± SEM, expressed as % control to combine the data for multiple (i.e., 9, 10, 4, 10, 14, and 14 for plasma, liver, aorta, intestine, heart, and fat tissue, respectively) species of EpFAs and diols (see Supplemental Table S1 for absolute levels/values of individual EpFAs, diols, and their ratios). *, *p* < 0.05; **, *p* < 0.01; ***, *p* < 0.001 vs. Pre.

**Figure 3:**
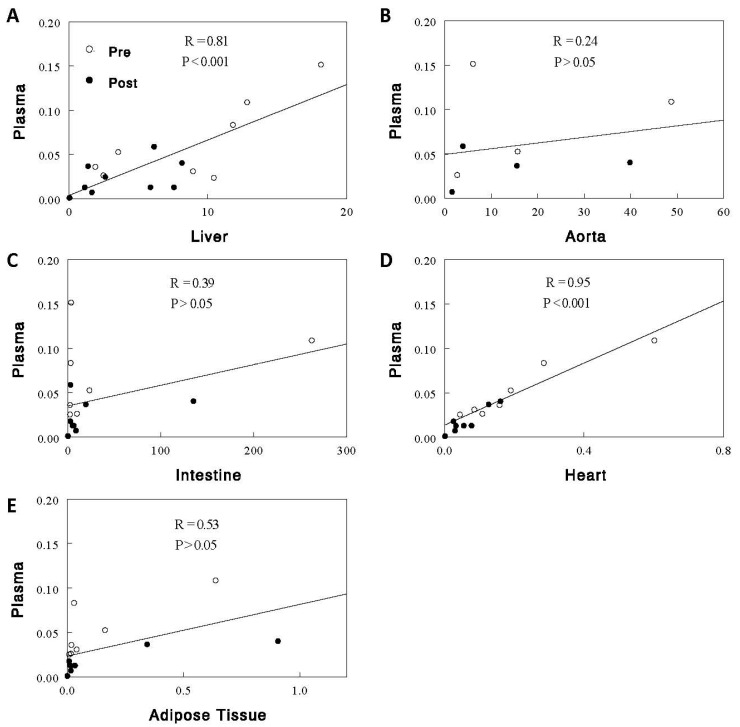
Correlation of D-to-E ratios between plasma and individual tissues ((**A**), liver; (**B**), aorta; (**C**), large intestine; (**D**), heart; (**E**), adipose tissue). Open and closed circles represent the ratios observed in the preprandial (Pre) and postprandial (Post) states, respectively. *p* values were adjusted for multiple comparisons using the Bonferroni method.

Circulating EpFA and diol levels, expressed as % control to combine the data on multiple species of EpFAs and diols, decreased after a 2.5 h feeding (Figure 2) with more profound decreases in diols (~70%) than EpFAs (~42%), resulting in significant decreases in D-to-E ratios (~54%, *p* < 0.001 for all). These changes were accompanied by similar changes in the liver but not in other tissues. In the liver, EpFAs, diols, and D-to-E ratios decreased after the feeding by 18% (*p* < 0.05), 55% (*p* < 0.001), and 36% (*p* < 0.01), respectively. In the aorta, EpFA levels increased by ~17% (*p* < 0.001), rather than decreased, and diol levels were unaltered, resulting in a small (~24%, *p* < 0.05) decrease in D-to-E ratios. In the intestine, neither EpFA/diol levels nor D-to-E ratios were altered by the feeding. In the heart, EpFA levels were unaltered, but diol levels and D-to-E ratios decreased (*p* < 0.01 for both). In adipose tissue, both EpFAs and diols decreased (*p* < 0.001 for both), but the degrees of these changes were similar, resulting in no significant changes in D-to-E ratios. Thus, the changes in circulating EpFAs, diols, and D-to-E ratios were best matched by those in the liver, suggesting that the liver may be the major tissue responsible for these changes in plasma. A strong correlation of D-to-E ratios in plasma was observed with those in the liver (*R* = 0.81, *p* < 0.001; Figure 3). Interestingly, this correlation was even stronger between plasma and the heart (*R* = 0.95, *p* < 0.001; see Section 3).

### 2.2. Effects of Acute Feeding on Renal Excretion of EpFAs and Diols

**Figure 4:**
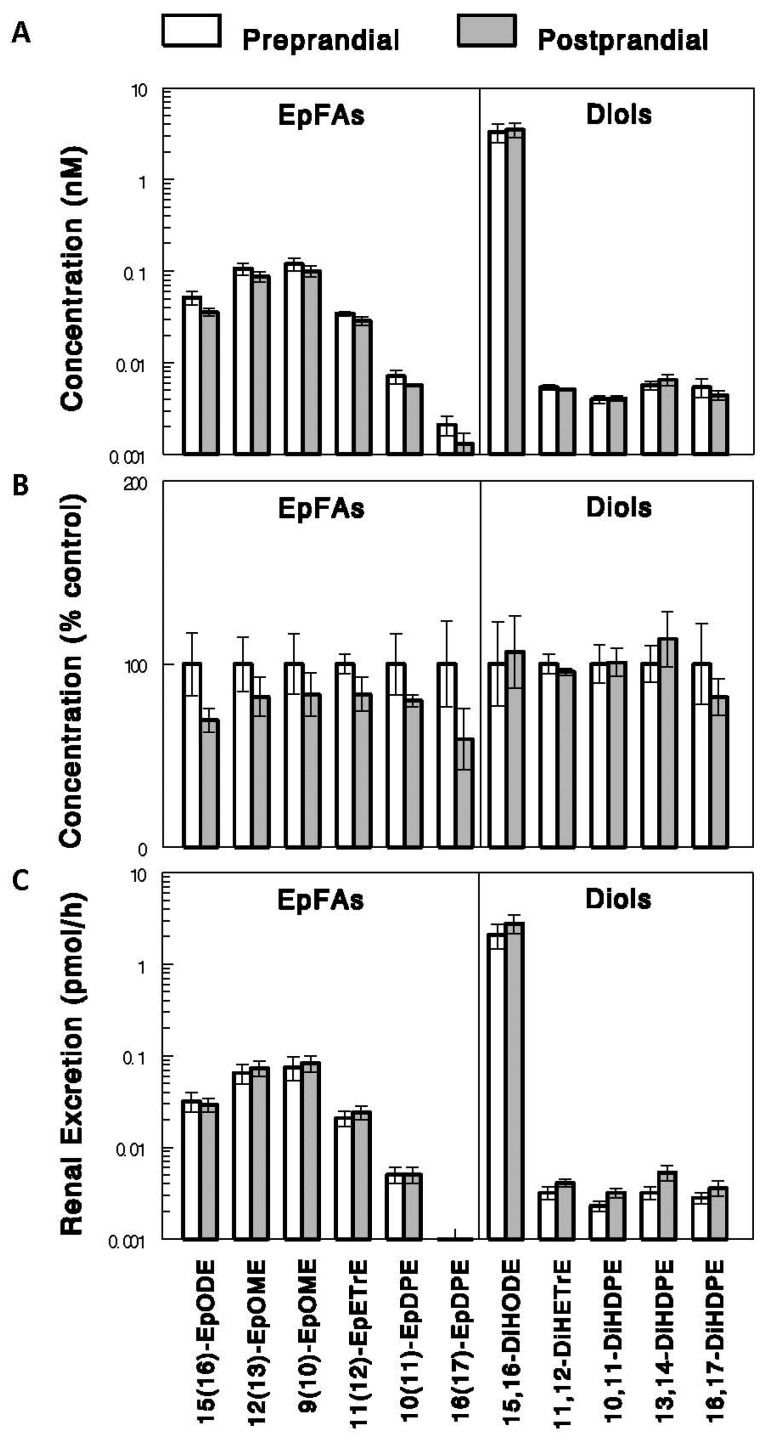
Urine levels (**A**,**B**) and renal excretions (**C**) of EpFAs and diols. EpFA and diol levels, measured in the urine collected before (preprandial state) and after (postprandial state) acute 2.5 h feeding in rats are shown as absolute concentrations (**A**) and as % control (i.e., preprandial; (**B**)). Renal excretions were calculated as urine concentrations multiplied by urine flow rates. Data show means ± SE (*n* = 5 or 6).

Numerous EpFAs and diols were detected in the urine (Figure 4). The 2.5 h feeding altered neither EpFA nor diol levels in the urine (*p* > 0.05 for all). In addition, urine volumes were not altered by acute feeding (0.62 ± 0.08 and 0.79 ± 0.08 mL/h before and after feeding, respectively; *p* > 0.05). The renal excretions of EpFAs and diols, calculated as urine concentrations of EpFAs and diols multiplied by urine flow rates, were not altered by the feeding (*p* > 0.05 for all), providing evidence against the possibility that renal excretions of EpFAs and diols play a role in altering plasma EpFAs, diols, or D-to-E ratios, at least under the condition of acute feeding. EpFA and diol levels in the urine were substantially lower than those in the plasma, especially in the preprandial states (<1% for all EpFAs and <5% for all diols except 15,16-DiHODE, the urinary levels of which were 32% plasma levels (see Section 3); Supplemental Table S2).

### 2.3. Effects of Diets on Circulating EpFAs, Diols, and D-to-E Ratios

**Figure 5:**
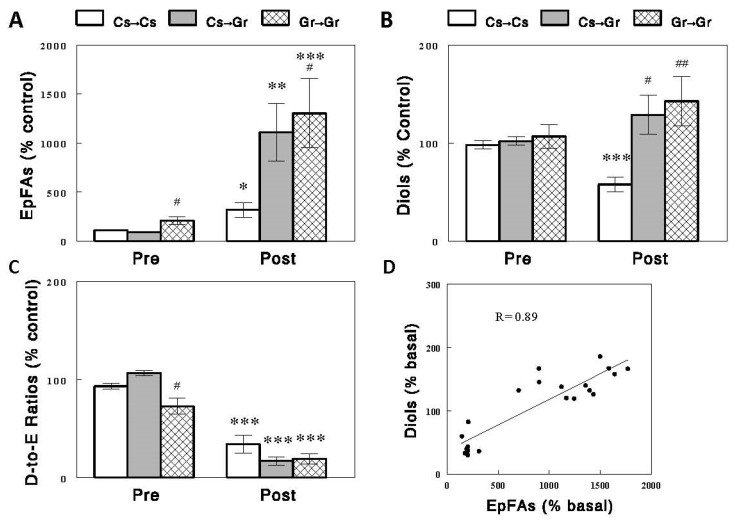
Effects of diets on plasma EpFAs (**A**), diols (**B**), and D-to-E ratios (**C**) in the preprandial (Pre) and postprandial (Post) states and correlation (**D**) between postprandial changes (% Pre) in EpFAs and diols observed in all groups. In (**A**–**C**), data show means ± SEM (*n* = 7), expressed as % control (i.e., preprandial levels of chronic Cs-diet groups, Cs → Cs and Cs → Gr) to combine the data on multiple species of EpFAs and diols (see Supplemental Table S3 for absolute levels/values of individual EpFAs, diols, and their ratios). Cs → Cs, rat group maintained on the casein (Cs)-based purified diet for a week and acutely fed with the same Cs diet; Cs → Gr, rat group maintained on the Cs diet for a week and acutely fed with the grain (Gr)-based diet (i.e., rat chow); Gr → Gr, rat group maintained on the Gr diet for a week and acutely fed with the same Gr diet; #, *p* < 0.05; ##, *p* < 0.01 vs. Cs → Cs group (ANOVA followed by ad hoc analysis using the Bonferroni method); *, *p* < 0.05; **, *p* < 0.01; ***, *p* < 0.001 vs. Pre (paired *t*-test).

**Figure 6:**
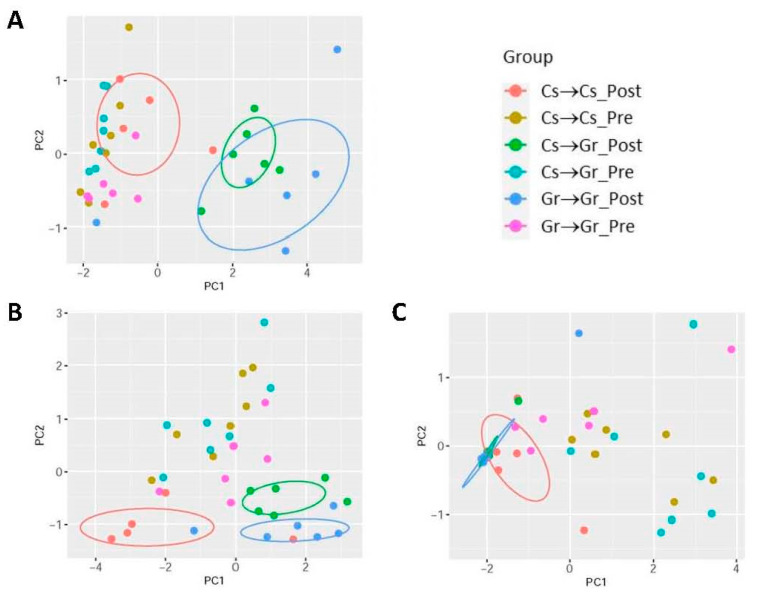
PCA plots of preprandial (Pre) and postprandial (Post) plasma EpFAs (**A**), diols (**B**), and D-to-E ratios (**C**). Circles were drawn to include 50% best representants around the centroid (i.e., average individual) for each cluster (or group).

Maintaining rats on the grain (Gr)-based diet for a week (chronic Gr-diet group), compared with those on the casein (Cs)-based purified diet (chronic Cs-diet group), increased plasma EpFA levels in the preprandial states (*p* < 0.05, Figure 5A, Pre) with no effects on FFA diol levels (Figure 5B), resulting in small decreases in D-to-E ratios (*p* < 0.05, Figure 5C). Acute (i.e., 2.5 h) feeding (Post) with the Cs diet in the chronic Cs-diet group (Cs → Cs) increased EpFA levels by ~3-fold (*p* < 0.05). In contrast, acute feeding with the Gr diet in the chronic Cs-diet and Gr-diet groups (Cs → Gr and Gr → Gr groups) increased EpFA levels by ~11- and ~13-fold, respectively (*p* < 0.01 for both), compared to the preprandial levels of the chronic Cs-diet group (i.e., Cs → Cs and Cs → Gr groups). Thus, the increases in plasma EpFAs were greater after acute feeding with the Gr diet than with the Cs diet, suggesting that the Gr diet may contain higher contents of EpFAs. This was confirmed by direct measurements of oxylipins in the diets (Supplemental Table S4). Higher EpFA levels after acute feeding with the Gr diet vs. Cs diet were associated with higher postprandial diol levels (*p* < 0.05, Figure 5B), and a strong correlation was observed between postprandial changes in EpFAs and diols in all groups (*p* < 0.001; Figure 5D). Despite the dramatic differences in postprandial changes in EpFAs and diols, the D-to-E ratios decreased similarly after acute feeding with the Cs vs. Gr diet (*p* < 0.001, Figure 5C), indicating that the D-to-E ratio may not be affected by EpFA levels or substrate effects (see Discussion). PCA analysis of EpFAs, diols, and D-to-E ratios (Figure 6) shows that the groups acutely fed with the Gr diet (Cs → Gr and Gr → Gr groups) were well separated from the group fed with the Cs diet (Cs → Cs group) with respect to postprandial EpFAs and diols. In contrast, all three diet groups were not separated from each other with respect to postprandial D-to-E ratios. These results are consistent with the data on postprandial EpFA and diol levels and D-to-E ratios (Figure 5).

### 2.4. Effects of Diets on Circulating Metabolic Parameters

**Figure 7:**
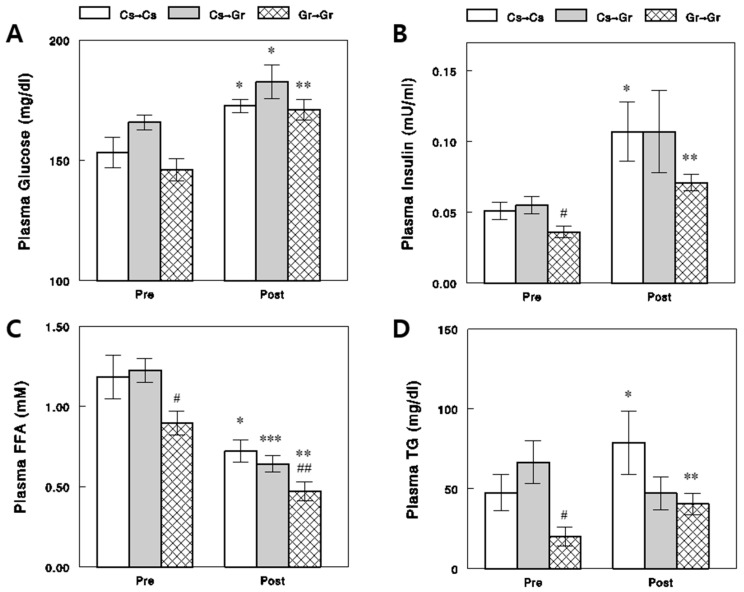
Effects of diets on plasma glucose (**A**), insulin (**B**), FFA (**C**), and TG (**D**) in the preprandial (Pre) and postprandial (Post) states. Data show means ± SEM (*n* = 7). #, *p* < 0.05; ##, *p* < 0.01 vs. chronic Cs-diet groups (Cs → Cs and Cs → Gr; unpaired *t*-test); *, *p* < 0.05; **, *p* < 0.01; ***, *p* < 0.001 vs. Pre (paired *t*-test).

**Figure 8:**
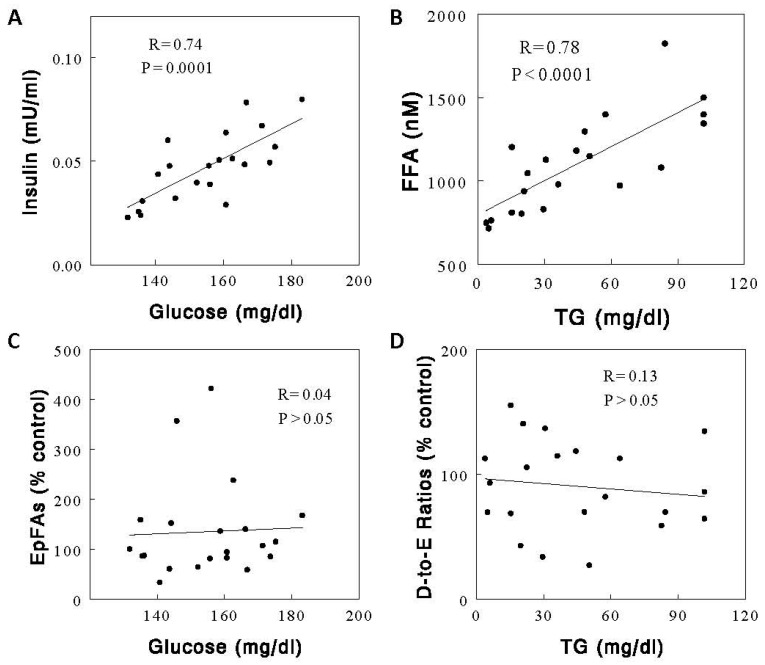
Correlations between metabolic parameters, i.e., glucose and insulin (**A**) and TG and FFA (**B**) and between metabolic parameters and EpFAs (**C**) or D-to-E ratios (**D**) in the preprandial states of all diet groups (*n* = 21).

We found plasma insulin, FFA, and triglyceride (TG) levels in the preprandial states were significantly lower in the chronic Gr-diet than the chronic Cs-diet group (Figure 7, *p* < 0.05 for all). Plasma glucose levels were also lower in the Gr-diet than in the Cs-diet group (*p* = 0.05). The insulin resistance index HOMA-IR, calculated as (glucose [mg/dL] × insulin [μU/mL] ÷ 405 [22]), was lower by ~38% in the Gr-diet than the Cs-diet group (13.1 ± 1.9 vs. 21.1 ± 2.1, *p* < 0.05). Thus, compared to the Gr-diet group, the Cs-diet group showed slightly insulin-resistant states (see Section 3). There were strong correlations among these metabolic parameters in the preprandial states (e.g., between glucose and insulin or between TG and FFA; Figure 8A,B), but none of these metabolic parameters explained the changes induced by the diets in plasma EpFAs and D-to-E ratios in the preprandial states (Figure 5); no significant correlations were observed between changes in the metabolic parameters vs. plasma EpFAs, diols, or D-to-E ratios (Figure 8C,D). Plasma glucose and insulin levels increased, and plasma FFA levels decreased after acute feeding, and these patterns were similar between the diet groups (Figure 7). Again, none of these changes in the metabolic parameters accounted for the changes in plasma EpFAs, diols, and D-to-E ratios in the postprandial states (see Supplemental Table S5 for correlations for all different combinations of parameters).

## 3. Discussion

The present study demonstrates that postprandial changes in plasma EpFA and diol levels and their ratios were very similar to those observed in the liver but not in other tissues, suggesting that the liver is responsible for changes in plasma EpFAs, diols, and D-to-E ratios and that plasma D-to-E ratios may reflect those in the liver, which may in turn represent sEH activities in the liver. In addition, we show that EpFAs and diols are excreted to the urine, but their levels were not significantly altered after a meal, suggesting that renal excretion of EpFAs, diols, and their conjugates may not significantly affect postprandial changes in circulating EpFA and diol levels or D-to-E ratios. Furthermore, we show that the diets, especially the grain-based chow diet, contained EpFAs and diols, and diet intake had a significant impact on circulating EpFAs and diols. Interestingly, the effects of these diets on circulating EpFAs and diols did not significantly alter postprandial changes in D-to-E ratios, suggesting that these changes in D-to-E ratios, reflecting sEH activities, may not be significantly affected by the availability of sEH substrates (i.e., EpFAs), as directly demonstrated by our previous study [23].

It is not surprising that the liver has a strong influence on plasma oxylipins, especially considering its capacity to generate EpFAs and diols; the liver has high specific activities of CYP and sEH enzymes [1,3]. In addition, the liver is a relatively large organ heavily perfused through the hepatic portal system, and the liver sinusoid (capillary) has an endothelial lining different from that of other tissues, due to the presence of open pores or fenestrae and high endocytotic capacity [24]. These characteristics likely facilitate the rapid exchange of metabolites or substances between the blood and hepatic parenchymal cells. Our data suggest that plasma EpFA and diol levels, in particular D-to-E ratios, reflect those in this large metabolically active organ; we found a strong correlation in the D-to-E ratios between plasma and the liver. The R value of 0.81 for this correlation indicates that the majority (i.e., 66% [=0.81^2^]) of the variations in the ratios in plasma was accounted for by variations in the liver. 

Although a strong correlation was observed in D-to-E ratios between plasma and the liver, the ratios were much greater in the liver (also in other tissues) than in plasma. Higher D-to-E ratios may arise from higher diol levels and/or lower EpFA levels. Diol levels were not much different between plasma and the liver, but EpFA levels were much (>50 times) greater in plasma than in the liver (see Supplemental Table S1). If EpFAs are produced mainly in the liver, how is it that EpFA levels are higher in plasma than in the liver? Is it because sEH, a major enzyme for the degradation of EpFAs, has high specific activity in the liver but not in plasma so that EpFAs rapidly disappear in the liver in vivo or in vitro (during sample collection and processing) but not so in plasma to maintain higher contents? Interestingly, despite the large differences in D-to-E ratios between plasma and the liver, the D-to-E ratios correlate very strongly between plasma and the liver, such that any changes in plasma D-to-E ratios (e.g., after a meal) well reflect those in the liver.

We found postprandial decreases in D-to-E ratios, reflecting sEH activities, occurred not only in the liver, but also in the heart. This observation may suggest a mechanism(s) for postprandial sEH inhibition that operates in multiple tissues. Alternatively, it is possible that sEH inhibition and decreases in D-to-E ratios occur in one of these tissues, causing similar changes in plasma, which in turn affect oxylipins in the other tissue. Because correlation does not reveal cause–effect relationships, we cannot say with confidence which tissue is primarily responsible. However, based on similar changes not only in D-to-E ratios but also in EpFA and diol levels between plasma and the liver (Figure 2), together with the hepatic capacities to generate EpFAs and diols and exchange metabolites with plasma, as discussed above, we believe the liver may be the tissue driving all these changes. Similar conclusions were recently drawn based on studies with hepatocyte selective sEH knockouts or sEH reduction associated with brain injuries [25,26]. 

Our data show that multiple species of EpFAs and diols were detected in the urine, but their concentrations were much lower compared to those in plasma. EpFAs and diols may appear in the urine through multiple mechanisms, such as glomerular filtration, glucuronidation, and sulfation. In plasma, most EpFAs and diols may bind to albumin, fatty acid-binding proteins, and lipoproteins, and unbound EpFAs and diols may account for very small fractions of the total plasma pools. There are also the rapid uptake (esterification) and release of EpFAs and possibly diols from fat and cholesterol in lipoproteins. Likely, only unbound but not bound or esterified EpFAs and diols are filtered through the glomerulus although the free and bound (or esterified) are certainly in dynamic equilibrium. In addition, the EpFAs are highly lipophilic and even the far more polar diols are likely subject to reuptake in renal tubules. If so, this may explain the lower concentrations of EpFAs and diols in the urine, compared to plasma levels. On the other hand, EpFAs and diols may be converted to glucuronides or sulfated forms to be more soluble and thus excreted in the urine. The low concentrations of EpFAs and diols in the urine indicate that these means of EpFA and diol excretion may not be the major contributors. Interestingly, one diol, 15,16-DiHODE, showed much higher concentrations in the urine, compared with other diols; its concentration in the urine was ~30% of plasma levels in preprandial states, whereas all the other diols showed values lower than 5%. It is possible that urinary EpFAs and diols are derived mainly from the kidney cells [27], rather than the filtration or conjugations discussed above. If so, the high concentrations of 15,16-DiHODE in the urine may reflect those in the kidney. Anyway, an important observation in the present study is that the urinary excretions of EpFAs and diols were not altered by acute feeding, suggesting that the kidneys may not play a major role in altering postprandial changes in circulating EpFAs, diols, and D-to-E ratios.

In the present study, we examined the effects of two different diets previously used as a “normal” or control diet in different studies [21,28]. We found these diets contained different amounts of EpFAs and diols (see Supplemental Table S4). Our data certainly support the practice of always monitoring the lipid composition of diets in such studies. The grain-based natural diet (Gr diet) had higher contents of EpFAs and diols, compared to the purified diet (Cs diet). Because EpFAs and diols are abundant in aerobic organisms, including plants, it makes sense that the grain-based diets contained substantial amounts of EpFAs and diols. In contrast, purified diets are made up with purified ingredients, and oxylipins may be eliminated in their purification processes. Also, many diets are cooked during processing into pellets which will lead to the oxidation of unsaturated fatty acids and particularly the peroxidation of polyunsaturated fatty acids which can rearrange to toxic unsaturated aldehydes. Our findings have important implications for studies of the dietary effects on oxylipins as mediators of various cellular processes [1,2,3]; if different diets are employed to study dietary effects on circulating oxylipins, it will be important to control and carefully monitor both free fatty acids and oxylipin levels in the diets, especially when comparing purified (e.g., high-fat diet) vs. grain-based (regular chow) diets.

Diet intake had significant impacts on circulating oxylipins. In particular, acute feeding with both the grain-based and the purified diets increased circulating EpFAs; these effects were greater with the grain-based than with the purified diet, probably because the dietary contents of EpFAs were greater in the grain-based diet. In contrast, feeding with these diets did not increase FFA diols despite the significant dietary contents of FFA diols. In fact, postprandial FFA diol levels decreased after feeding with the purified diet, suggesting a postprandial reduction in the sEH enzyme (thus less conversion of EpFAs to diols). Thus, dietary FFA diols seemed not to have much impact on circulating diols in contrast with the profound effects of dietary EpFAs to increase circulating EpFAs. This difference may be explained by the differences in the absorption or stabilities of EpFAs vs. diols in acidic conditions of the stomach. Although high in a few plants, EpFAs are very minor dietary components. EpFAs are relatively stable to hydrolysis and may survive in the acidic stomach by forming lipid micelles to stay intact until they reach the intestine to be absorbed into the circulation. In contrast, FFA diols are highly polar and may not be protected in lipid micelles, and a significant proportion of them may be chemically hydrolyzed in the stomach. In addition, the more lipophilic EpFAs would be expected to be absorbed far more readily than diols.

We found the animals maintained on the two “normal” diets (i.e., Gr and Cs diets) showed different metabolic parameters in the preprandial states; plasma insulin, FFA, and TG levels were significantly lower in the Gr- than in the Cs-diet group. The Cs-diet (i.e., purified diet) group showed slightly insulin-resistant states, reflected in the HOMA-IR index, possibly due to the high (i.e., 35%) sucrose content of the diet. Significant correlations were observed between metabolic parameters (e.g., between FFA and TG or between insulin and glucose) in all groups (see Supplemental Table S5). We also observed significant correlations between EpFA and diol levels. However, none of the metabolic parameters correlates with changes in EpFAs, diol, or D-to-E ratios, suggesting a factor(s) other than these metabolic parameters is responsible for changes in oxylipin metabolism. Thus, the profound decreases in the D-to-E ratios in the postprandial states may not be explained by plasma glucose, insulin, FFA, or TG, consistent with our previous observations [21,23]. Regarding this, it is interesting to note that hepatic sEH activity, measured in the liver homogenates, was not altered by acute feeding (Oh et al. unpublished data). If the postprandial decreases in D-to-E ratios indicate hepatic sEH activity in vivo, these data suggest that sEH activity may be inhibited by an inhibitor, possibly produced by gut bacteria, as this effect is absent in rats with gut bacteria removed by antibiotic treatment [21]. This intriguing possibility needs to be directly tested in future studies. Hepatic-sEH activity was decreased without changes in sEH proteins during brain injuries [26]. 

There are some limitations in the present study. The oxylipin assay is very expensive and time-consuming, and we were able to study only a few tissues. The brain was not included because fat uptake by the brain is slow and unlikely to account for postprandial changes that occur within 2.5 h after feeding. The kidney was not included; we chose instead to study the urinary excretions of epoxides and diols. Kidney cells may contribute to postprandial changes in circulating oxylipin levels, independent of their modulation of urinary oxylipin excretions. Finally, the study design for the diet effect study would be better if it employed a 2 × 2 factorial design, including a Gr → Cs group. However, with the practical limitations in the total number of assays, we chose to have more samples per group with the current design (i.e., three groups) rather than having more groups (i.e., four groups in the 2 × 2 design) at the expense of group sample sizes. Despite the less optimal design, thanks to the increased group sample sizes, the present data support our conclusions concerning the effects of dietary oxylipins on their circulating levels. 

In conclusion, the present data suggest that the liver is responsible for changes in plasma EpFAs, diols, and D-to-E ratios and that plasma D-to-E ratios, often used as indices of sEH activity, may reflect those in the liver. The present data also suggest that renal excretion of EpFAs and diols were not significantly altered by feeding and may not play a role in postprandial changes in circulating EpFA and diol levels or D-to-E ratios. Finally, we show that diet intake had a significant impact on circulating EpFAs and diols, providing important implications for studies of dietary effects on oxylipins; it may be important to control dietary oxylipin levels in such studies, especially when comparing purified vs. grain-based natural diets.

## 4. Materials and Methods

### 4.1. Animals and Catheterization

Male Wistar rats weighing 280–300 g (approximately 9 weeks old) were obtained from Envigo (Indianapolis, IN, USA) and studied at least 5 days after arrival for acclimatization to the new environment. Animals were housed under controlled temperature (22 ± 2 °C) and lighting (12 h light, 6 a.m.–6 p.m.; 12 h dark, 6 p.m.–6 a.m.) with free access to water and food. Blood samples were obtained using a tail artery catheter in conscious states. At least 4 days before the experiment, the animals were placed in individual cages with tail restraints, as previously described [21], which was necessary to protect the tail artery catheter after its placement a few hours before the experiment (~12 p.m.). The animals were free to move about and were allowed unrestricted access to food and water. All procedures involving animals were approved by the Institutional Animal Care and Use Committee at the University of Southern California.

### 4.2. Animal Experiments

Effects of acute feeding (Study 1): Animals were fed with a gel diet prepared from a K^+^-deficient powdered diet (TD.88239; Envigo), as in our previous study [21], with supplementation with KCl to have normal 1% K^+^ content. Animals were acclimatized to the gel diet for 3–4 days before this acute feeding experiment. Blood samples were collected through the tail-artery catheter immediately before (preprandial state) or 2.5 h after the normal initiation of feeding (postprandial state; *n* = 6 each) at 6 PM when lights were off. Blood samples were rapidly spun, and plasmas were isolated; plasmas for oxylipin analysis were mixed with triphenylphosphine (TPP; 4 µg/mL), butylated hydroxytoluene (BHT; 4 µg/mL), and EDTA (20 µg/mL) [29]. TPP was used to reduce peroxides to their monohydroxy equivalent, and BHT was used to quench radical catalyzed reactions. Following blood sampling, the animals were anesthetized with isoflurane, and tissue samples (liver, fat, heart, aorta, and intestine) were collected after whole body perfusion with saline (~60 mL) to avoid the contamination of tissue samples with blood. Liver and fat tissues were selected as major tissues of lipid metabolism and storage/mobilization. The heart was selected as a major lipid-metabolizing tissue, and the aorta to study the contributions of endothelium and vascular smooth muscle. Intestine (the large intestine) was included to study potential influences of gut bacteria on postprandial changes in sEH activity [21] (see Discussion for limitations). Urine passed was collected from the bottom of cages, as previously described [28], for 5 h before and 2.5 h during the feeding. Plasma, urine, and tissue samples were frozen immediately in liquid N_2_ and stored at −80 °C until analysis.

Diet effects (Study 2): Animals were maintained on a casein-based purified diet (TD.08267; Envigo; Cs diet) or grain-based natural diet (i.e., standard rat chow; Gr diet) for a week. After this, the animals maintained on the Cs diet were acutely fed with the same diet (Cs → Cs group) or the Gr diet (Cs → Gr group), and the animals maintained on the Gr diet were acutely fed with the same diet (Gr → Gr group). The feeding started at 6 PM, the normal feeding start time when lights are off, and blood samples were collected through the tail-artery catheter before and 2.5 h after the start of the feeding (*n* = 7 each), as in Study 1. Blood samples were rapidly spun, and plasmas were isolated, mixed with TPP, BHT, and EDTA (for oxylipin analysis), and frozen immediately in liquid N_2_ and stored at −80 °C until analysis. 

### 4.3. Assays

Plasma, urine, and tissue samples were analyzed for oxylipins using procedures previously reported [29]. Plasma glucose was analyzed using a glucose oxidase method on a GM9 Glucose Analyzer (Analox Instruments, Stourbridge, UK). Plasma insulin was determined using a Rat Ultrasensitive Insulin ELISA kit from ALPCO (Salem, NH, USA). Plasma FFA levels were measured using an acyl-CoA oxidase-based colorimetric kit from Wako Chemicals Inc. (Richmond, VA, USA). Plasma triglyceride (TG) levels were analyzed using a Ponte Scientific TG reagent (Thermo Fisher Scientific; Waltham, MA, USA).

### 4.4. Statistical Analysis

All data are expressed as means ± S.E.M. The significance of differences in the mean values were assessed by student’s *t*-tests or one-way ANOVA (assuming normality of the data) followed by ad hoc analysis using the Bonferroni method for multiple comparisons. The significance of correlation was assessed by the Pearson’s Method adjusted with Bonferroni’s factor for multiple comparisons. A *p* value less than 0.05 was considered to be statistically significant. Principal component analysis (PCA) was performed to summarize the variation of the variables in the original dataset in parsimonious set of uncorrelated variables called principal components where each of them is a linear combination of all of the original variables. The new set of principle components are derived in decreasing order of importance so that the first principal component accounts for the highest proportion of the variance. There are a couple of objectives for conducting this analysis. First, PCA is useful for identifying the smallest set of principal components that will capture the majority of the variance of the dataset. Second and final, the principal components can be displayed in a pairwise fashion on a scatter plot. The purpose is to visually identify possible clustering of the data when certain characteristics of the original dataset are considered.

## Data Availability

Data are contained within the article and supplementary materials.

## Supplementary Materials

The supporting information can be downloaded at: https://www.mdpi.com/article/10.3390/ijms242417351/s1.
